# Step-Wise Increase in Tigecycline Resistance in *Klebsiella pneumoniae* Associated with Mutations in *ramR*, *lon* and *rpsJ*

**DOI:** 10.1371/journal.pone.0165019

**Published:** 2016-10-20

**Authors:** Li Fang, Qiong Chen, Keren Shi, Xi Li, Qiucheng Shi, Fang He, Jiancang Zhou, Yunsong Yu, Xiaoting Hua

**Affiliations:** 1 Department of Infectious Diseases, Sir Run Run Shaw Hospital, College of Medicine, Zhejiang University, Hangzhou, Zhejiang, People's Republic of China; 2 State Key Laboratory for Diagnosis and Treatment of Infectious Disease, First Affiliated Hospital, College of Medicine, Zhejiang University, Hangzhou, Zhejiang, People's Republic of China; Cornell University, UNITED STATES

## Abstract

*Klebsiella pneumoniae* is a gram-negative bacterium that causes numerous diseases, including pneumonia and urinary tract infections. An increase in multidrug resistance has complicated the treatment of these bacterial infections, and although tigecycline shows activity against a broad spectrum of bacteria, resistant strains have emerged. In this study, the whole genomes of two clinical and six laboratory-evolved strains were sequenced to identify putative mutations related to tigecycline resistance. Of seven tigecycline-resistant strains, seven (100%) had *ramR* mutations, five (71.4%) had *lon* mutations, one (14.2%) had a *ramA* mutation, and one (14.2%) had an *rpsJ* mutation. A higher fitness cost was observed in the laboratory-evolved strains but not in the clinical strains. A transcriptome analysis demonstrated high expression of the *ramR* operon and *acrA* in all tigecycline-resistant strains. Genes involved in nitrogen metabolism were induced in the laboratory-evolved strains compared with the wild-type and clinical strains, and this difference in nitrogen metabolism reflected the variation between the laboratory-evolved and the clinical strains. Complementation experiments showed that both the wild-type *ramR* and the *lon* genes could partially restore the tigecycline sensitivity of *K*. *pneumoniae*. We believe that this manuscript describes the first construct of a *lon* mutant in *K*. *pneumoniae*, which allowed confirmation of its association with tigecycline resistance. Our findings illustrate the importance of the *ramR* operon and the *lon* and *rpsJ* genes in *K*. *pneumoniae* resistance to tigecycline.

## Introduction

*Klebsiella pneumoniae* is a gram-negative bacterium of the Enterobacteriaceae family [1] that can cause numerous diseases, including pneumonia, urinary tract infections, septicemia, and pyogenic live abscesses [2]. Resistance of *K*. *pneumoniae* to carbapenems is increasing worldwide [3], and this rise in multidrug resistance has limited the available treatment options for this bacterium, which currently include only colistin, tigecycline, aminoglycosides, and fosfomycin [4]. Moreover, strains resistant to tigecycline have been reported [5–8].

Tigecycline belongs to the glycylcycline family of antibiotics, which consists of drugs modified from minocycline, and has bacteriostatic activity against a broad spectrum of gram-positive and gram-negative bacteria [9]. Compared with tetracycline, tigecycline exhibits increased affinity for the ribosome due to its interaction with 16S rRNA, and this increased affinity proves helpful for overcoming TetM-mediated resistance [10]. Resistance to tigecycline is mainly attributed to overproduction of the AcrAB-TolC efflux pump, which is regulated by RamA in *K*. *pneumoniae* [7]. *ramA* transcription is de-repressed by the *ramR* mutation in *K*. *pneumoniae* [5], and both *rarA* and *marA* provide alternate pathways for RamA-independent tigecycline resistance [11]. Moreover, a mechanism for tigecycline resistance independent of the AcrAB-TolC pump has also been identified; mutations in *rpsJ* encoding ribosomal protein S10 and *kpgABC* encoding a putative transporter are associated with AcrAB-TolC-independent tigecycline resistance [6, 8].

In this study, we combined whole-genome sequencing and RNA-Seq to identify putative mutations related to tigecycline resistance in both clinical and laboratory-evolved strains of *K*. *pneumoniae*. Mutations in the *ramR*, *lon*, *ramA* and *rpsJ* genes were observed in the tigecycline-resistant strains. In addition, the fitness costs associated with the mutants were detected to predict the risk of the bacteria spreading in the environment. A transcriptome analysis demonstrated that the *ramR* locus was highly expressed in all tigecycline-resistant strains. To confirm the role of *ramR* and *lon* in tigecycline resistance in *K*. *pneumoniae*, we performed a complementation experiment and constructed a knockout strain.

## Materials and Methods

### Bacterial isolates and antimicrobial susceptibility testing

The bacteria evaluated in this study included the clinical isolates XH209 and XH210 [12], the laboratory-evolved mutants XH211-XH216 and two gene-knockout mutants (*ramR* XH872 and *lon* XH889) of *K*. *pneumoniae* (Table 1). Strain XH209 was isolated from the blood of a patient in Hangzhou China who was at the beginning of tigecycline treatment, and a strain isolated after the patient received tigecycline treatment was denoted XH210. The MICs were determined by broth microdilution with cation-adjust Mueller-Hinton (MH) broth or by Etest (bioMérieux, Marcy l'Etoile, France) on MH agar, and the results were interpreted according to the CLSI or EUCAST breakpoints. The bacteria were cultured in Luria-Bertani (LB) or MH (Oxoid, Basingstoke, UK) medium at 37°C. Hygromycin and apramycin were added to the media to final concentrations of 100 mg/L and 50 mg/L, respectively, as necessary.

**Table 1:** Strains and plasmids used in this study.

Strain/plasmid	Isolate day	Parental strain	genotype	Other genetic changes	Reference
XH209	0	NA	NA	NA	this study
XH210	1	XH209	*ramR* Q122*	NA	this study
XH211	13	XH209	*ramA* Q72L, *lon* Q317* *ramR* Δ190 bp (322–511)	*cspE* N57K	this study
XH212	13	XH209	P*ramR* +G, *lon* D445V, *rpsJ* V57L	*tetA* I235F, 300 kb dup	this study
XH213	13	XH209	*ramR* A40T, *lon* R33W, *rpoC* Δ18 bp (634–651)	*eutL* E95Q	this study
XH214	13	XH209	*ramR* L58P, *rpoC* G336A		this study
XH215	13	XH209	*ramR* Q135*, *lon* Δ 9 bp (791–799), *rpoC* S263Y	*yfiR* C89Y *hypo* K302T	this study
XH216	13	XH209	*ramR* S29*, lon N417K	Mobile element protein G12E	this study
XH490	1	XH209	*ramR* Q122*	ND	this study
XH491	1	XH209	*ramR* T42Ins (8 bp)	ND	this study
XH492	1	XH209	*ramR* S137*	ND	this study
XH493	1	XH209	*ramR* A49Ins (8 bp)	ND	this study
XH494	1	XH209	*ramR* M1V	ND	this study
XH495	1	XH209	*ramR* W185*	ND	this study
XH496	1	XH209	*ramR* F45Del (8 bp)	ND	this study
XH497	1	XH209	*ramR* A2FS	ND	this study
XH498	1	XH209	*ramR* F45Ins (8 bp)	ND	this study
XH499	1	XH209	*ramR* R107H	ND	this study
XH500	1	XH209	*ramR* W89L	ND	this study
XH501	1	XH209	*ramR* A37V	ND	this study
XH502	1	XH209	*ramR* W89*	ND	this study
XH503	1	XH209	*ramR* T119P	ND	this study
XH504	1	XH209	*ramR* K5FS	ND	this study
XH505	1	XH209	*ramR* A105G	ND	this study
XH466		XH210	XH210 /pCR2.1-T vector		this study
XH468		XH210	XH210 /pCR2.1-ramR		this study
XH539		XH211	XH211 /pCR2.1-T vector		this study
XH540		XH211	XH211 /pCR2.1-lon		this study
XH583		XH211	XH211 /pCR2.1-ramR		this study
XH585		XH211	XH211 /pCR2.1-lon-ramR		this study
XH541		XH212	XH212 /pCR2.1-T vector		this study
XH593		XH212	XH212 /pCR2.1-ramR		this study
XH542		XH212	XH212 /pCR2.1-lon		this study
XH587		XH212	XH212 /pCR2.1-lon-ramR		this study
XH396		XH213	XH213 /pCR2.1-T vector		this study
XH398		XH213	XH213 /pCR2.1-ramR		this study
XH579		XH213	XH213 /pCR2.1-lon		this study
XH589		XH213	XH213 /pCR2.1-lon-ramR		this study
XH448		XH214	XH214 /pCR2.1-T vector		this study
XH452		XH215	XH215 /pCR2.1-T vector		this study
XH450		XH214	XH214 /pCR2.1-ramR		this study
XH581		XH215	XH215 /pCR2.1-lon		this study
XH591		XH215	XH215 /pCR2.1-lon-ramR		this study
XH456		XH216	XH216 /pCR2.1-T vector		this study
XH454		XH215	XH215 /pCR2.1-ramR		this study
XH544		XH216	XH216 /pCR2.1-lon		this study
XH568		XH216	XH216 /pCR2.1-lon-ramR		this study
XH872		XH209	Δ*ramR*::apr		this study
XH889		XH209	Δ*lon*::apr		this study
XH478		XH209	XH209 /pACBSR-Hyg		this study
plasmid					
pCR2.1-T vector					Thermo Fisher Scientific
pCR2.1-ramR			pCR2.1-T vector carrying wild-type *ramR*		this study
pCR2.1-lon			pCR2.1-T vector carrying wild-type *lon*		this study
pCR2.1-lon-ramR			pCR2.1-T vector carrying wild-type *ramR* and *lon*		this study
pIJ773			Template for amplification of the apramycin resistance gene		Pep Charusanti
pACBSR-Hyg			A p15A replicon plasmid containing an arabinose-inducible λ-Red recombinase and hygromycin resistance selection marker		Pep Charusanti

Note:

*: stop codon;

Δ: Deletion; Ins: Insertion; Del: Deletion; FS: frame shift;

### Laboratory evolution of tigecycline-resistant mutants

Six independent single colonies of *K*. *pneumoniae* XH209 were grown overnight at 37°C, and the cultures were diluted in LB broth with a serially increasing concentration of tigecycline. The concentration of tigecycline was started at a value equal to 1/2 MIC and doubled every 24 h. The overnight cultures were stored at -80°C for further experiments and analysis [13].

The overnight cultures of *K*. *pneumoniae* XH209 were plated on LB plates containing 4 mg/L tigecycline. Mutants were randomly selected from the plates after incubation at 37°C for 24 h and then streaked onto LB plates. The colonies were stored in LB medium with 15% glycerol. The *ramR* gene of the mutants was amplified by PCR and Sanger sequencing [14].

### Homology modeling

RamR structure homology modeling was performed with the SWISS-MODEL workspace using the structure of RamR from *Salmonella Typhimurium* (PDB ID: 3VVX) as a template [15]. The 3D structure of the RamR protein was visualized using the PyMOL molecular graphics system, and the positions of the mutations were labeled with the corresponding amino acids.

### Whole-genome DNA sequencing and analysis

Bacteria from a single colony were cultured overnight at 37°C in MH broth. Genomic DNA was extracted using a QIAamp DNA Mini Kit (Qiagen, Valencia, CA, USA) following the protocol recommended by the manufacturer. Agarose gel electrophoresis and a NanoDrop spectrophotometer were used to determine the quality and quantity of the extracted genomic DNA, respectively. The 300-bp library used for Illumina paired-end sequencing was constructed using 5 μg of genomic DNA from the two clinical strains and six laboratory-evolved mutants. In addition, an 8-kb mate-pair library was prepared for XH209 to complete its genome [16]. The raw Illumina data were *de novo* assembled using IDBA-Hybrid [17]. The pre-assembled contigs were arranged into scaffolds using SSPACE [18], and gaps within the scaffolds were closed with GapFiller [19]. Mapping and SNP detection were performed using the CLC Genomics Workbench (CLC bio, Aarhus, Denmark). The regions containing the detected SNPs were amplified by PCR using the primers listed in S1 Table. The PCR products were sent to Biosune (Hangzhou, China) for Sanger sequencing.

### Growth rate measurement

Four independent cultures of each strain were grown overnight and diluted to 1:1000 in LB, and four replicates of each culture were aliquoted into a flat-bottom 96-well plate. The plate was incubated at 37°C with agitation, and the OD_600_ of each culture was determined every 5 min for 16 h using a BioTEK Synergy plate reader (BioTEK, Winooski, VT, USA). The growth rate was estimated based on the OD_600_ curves using R script [20].

### RNA-Seq and transcript analysis

The wild-type and mutant strains were grown overnight in 2 mL of LB broth at 37°C. The overnight cultures were diluted 1:100 in 50 mL of LB broth and incubated at 37°C with shaking for 2 h. The bacteria were pelleted at 4°C, and after grinding in liquid nitrogen, total RNA was extracted using TRIzol Reagent (Invitrogen, Carlsbad, CA, USA). Then, 10 U of RNase-free DNase I (Promega, Mannheim, Germany) was added to the samples, and the RNA was purified through phenol-chloroform extraction. The RNA quality and quantity were determined by 1.0% formaldehyde denaturing agarose gel electrophoresis and a NanoDrop ND-1000 spectrophotometer, respectively. rRNA removal and RNA sequencing were performed as previously described [21] by staff at Zhejiang Tianke (Hangzhou, China). The raw data from the samples were analyzed using Subread [22, 23], and the raw counts of each sample were normalized and processed using the EdgeR Bioconductor package [24]. Genes with adjusted p-values (BH method) less than 0.05 and presenting at least two-fold differences in expression were considered to be differentially expressed.

### Complementation experiment

Plasmids carrying wild-type *ramR* or *lon* were constructed and then introduced into laboratory-evolved resistant strains of *K*. *pneumoniae* by electroporation. Briefly, a region including the open reading frame of *ramR* or *lon*, derived from the XH209 genome sequence, was cloned into the pCR 2.1 vector (Invitrogen, USA). The plasmid containing the *ramR* gene and/or *lon* gene was then transferred into the resistant strains. The empty vector (pCR 2.1 vector) was also introduced into the resistant strains as a control. The MIC for tigecycline of the transformants were determined by broth microdilution with MH broth.

### Gene knockout

Mutant *ramR* and *lon* genes were constructed as previously described [25]. In brief, the pIJ773 plasmid was used as the template for amplification of an apramycin resistance cassette, and the pACBSR-Hyg plasmid was used for arabinose-inducible λ–Red recombination. The knockout cassette was amplified from the FRT-flanked Apra^R^ cassette of pIJ773 using response primers (Table 1). The PCR-amplified knockout cassette was then transformed into *K*. *pneumoniae* XH209+PACBSR-Hyg, and the transformants were screened overnight in LBApra at 37°C. The loss of pACBSR-Hyg was screened by streaking onto LBApra and low-salt LB + hygromycin plates overnight at 37°C. PCR and Sanger sequencing were performed to confirm the correct insertion of the knockout cassette.

## Results

### Clinical strains and *in vitro* selection of mutants with tigecycline resistance

**Fig 1:**
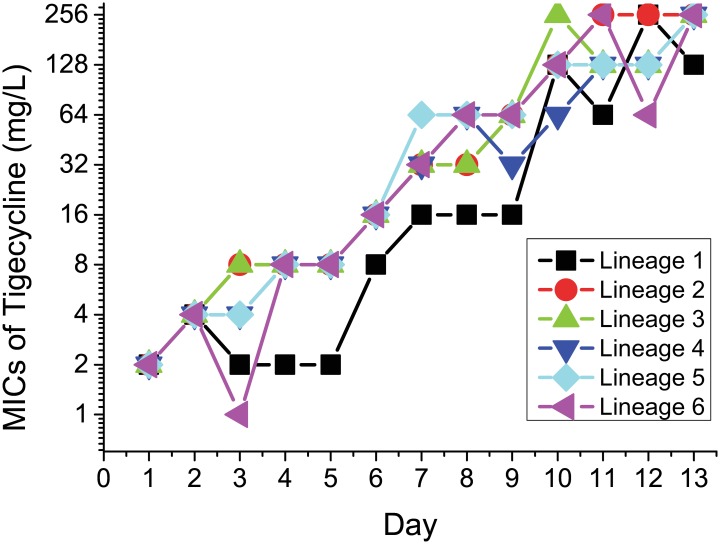
Resistance to tigecycline increased in a stepwise manner (as a fold increase over the MIC of *K*. *pneumoniae* XH209) following serial passage in tigecycline.

We obtained two *K*. *pneumoniae* strains that were isolated from the blood of a patient during tigecycline treatment. The *K*. *pneumoniae* tigecycline MICs increased from 2 mg/L (XH209) to 8 mg/L (XH210). Six independent colonies of XH209 were also selected at increased concentrations of tigecycline. After 13 days of serial passage (every 24 h), we obtained six tigecycline-resistant mutants, and the observed resistance to tigecycline increased in a step-wise manner (fold-increases compared with the MIC of *K*. *pneumoniae* XH209) following tigecycline passaging (Fig 1). The MICs of the six selected mutants ranged from 64 to 256 mg/L.

### Putative resistance mutations and their fitness costs

The whole genomes of the clinical isolates and *in vitro* selection mutants were sequenced to identify mutations that are potentially responsible for resistance to tigecycline. The most commonly observed SNPs were nucleotide substitutions resulting in amino acid changes or stop codons (Table 2). A mutation in *ramR* was found in seven strains. In *K*. *pneumoniae*, the *ramR* gene encodes a repressor of *ramA*, which is known to be associated with resistance to tigecycline and ciprofloxacin [5]. RamA is a positive global regulator of the AcrAB efflux system [14]. Five strains harbored a *lon* mutation, and a mutation in *rpoC* was detected in three strains. In addition, a 287-kb duplication was observed in the genome of *K*. *pneumoniae* XH212. The biological fitness costs of the clinical isolates and *in vitro* selection mutants were also measured based on their relative growth rates compared with that of XH209. Fitness costs ranging from 2% to 58% were observed in most of the strains, and the costs showed a good correlation with the lag time. Notably, the clinical isolates showed the lowest fitness costs.

**Table 2:** Characterization of laboratory-evolved tigecycline-resistant *K*. *pneumoniae* and single-step selection of *K*. *pneumoniae* mutants.

Strain	Parental strain	Putative mutation(s) causing reduced susceptibility to TGC	Other genetic changes	TGC MIC (mg/L)	TGC MIC (mg/L) +PaβN (50 mg/L)	Relative growth rate	Lag time (min)	TET	CHL	AK	CTX	CIP	IPM	NI
XH209	NA	NA	NA	2	2	1	120	>256	>256	2	>256	0.75	4	64
XH210	XH209	*ramR* Q122*	NA	16	8	0.98	113.44	>256	>256	2	>256	4	4	192
XH211	XH209	*ramA* Q72L, *lon* Q317* *ramR* Δ190 bp (322–511)	*cspE* N57K	128	8	0.42	232	>256	>256	1	1.5	3	0.125	3
XH212	XH209	P*ramR* +G, *lon* D445V, *rpsJ* V57L	*tetA* I235F, 300 kb dup	>256	256	0.59	183.75	>256	>256	1.5	>256	6	4	16
XH213	XH209	*ramR* A40T, *lon* R33W, *rpoC* Δ18 bp (634–651)	*eutL* E95Q	>256	64	0.46	223.44	>256	>256	0.5	128	3	4	8
XH214	XH209	*ramR* L58P, *rpoC* G336A		64	32	0.74	189.06	>256	>256	0.75	>256	2	1	128
XH215	XH209	*ramR* Q135*, *lon* Δ9 bp (791–799), *rpoC* S263Y	*yfiR* C89Y *hypo* K302T	256	64	0.56	187.19	>256	>256	0.75	64	3	2	4
XH216	XH209	*ramR* S29*, *lon* N417K	Mobile element protein G12E	64	16	0.55	241.25	>256	>256	0.75	128	2	6	4
XH490	XH209	*ramR* Q122*	ND	16	ND	0.98	123.75	>256	>256	1.5	>256	3	8	128
XH491	XH209	*ramR* T42Ins (8 bp)	ND	8	ND	0.98	120	>256	>256	1	>256	3	6	128
XH492	XH209	*ramR* S137*	ND	16	ND	0.98	113.44	>256	>256	1	>256	3	3	192
XH493	XH209	*ramR* A49Ins (8 bp)	ND	16	ND	0.97	232	>256	64	1	>256	3	3	>512
XH494	XH209	*ramR* M1V	ND	16	ND	0.99	183.75	>256	>256	1	>256	2	4	96
XH495	XH209	*ramR* W185*	ND	16	ND	0.97	223.44	>256	32	1.5	>256	4	6	128
XH496	XH209	*ramR* F45Del (8 bp)	ND	16	ND	0.88	189.06	>256	>256	1.5	>256	2	1	32
XH497	XH209	*ramR* A2FS	ND	8	ND	0.98	187.19	>256	>256	1.5	>256	2	6	64
XH498	XH209	*ramR* F45Ins (8 bp)	ND	16	ND	0.99	241.25	>256	>256	1.5	>256	2	3	128
XH499	XH209	*ramR* R107H	ND	16	ND	0.99	123.75	>256	>256	1.5	>256	2	3	64
XH500	XH209	*ramR* W89L	ND	16	ND	0.98	123.75	>256	>256	2	>256	3	2	96
XH501	XH209	*ramR* A37V	ND	8	ND	0.99	122.5	>256	>256	1.5	>256	3	4	128
XH502	XH209	*ramR* W89*	ND	8	ND	0.97	115.31	>256	>256	1.5	>256	3	6	128
XH503	XH209	*ramR* T119P	ND	8	ND	0.97	114.38	>256	>256	1.5	>256	3	4	192
XH504	XH209	*ramR* K5FS	ND	8	ND	0.98	123.44	>256	>256	1.5	>256	3	4	128
XH505	XH209	*ramR* A105G	ND	8	ND	0.97	124.06	>256	>256	1.5	>256	3	4	128

TGC: tigecycline; TET: tetracycline; CHL: chloramphenicol; AK: amikacin; CTX: cefotaxime; CIP: ciprofloxacin; IPM: imipenem;NI: nitrofurantoin. Note: NA: not found;ND: not detect;

*: Stop codon;

Δ: Deletion; Ins: Insertion; Del: Deletion; FS: Frameshift;

### Up-regulation of *the* ram locus in tigecycline-resistant mutants

In this study, we selected the genes exhibiting at least a two-fold change in expression level in the mutants compared with the wild-type XH209 strain. In total, seven (0.14%), 118 (2.42%), 82 (1.68%), 69 (1.41%), 55 (1.13%), 73 (1.50%) and 30 (0.61%) genes had increased expression in XH210, XH211, XH212, XH213, XH214, XH215 and XH216, respectively. Two (0.04%), 44 (0.90%), 152 (3.11%), 47 (0.96%), 87 (1.78%), 41 (0.84%) and 78 (1.60%) genes showed reduced expression in these strains, respectively. The up-regulation of seven genes was observed in all seven strains. The annotations and reads per kilobase per million mapped reads (RPKM) values are listed in Table 3. These genes can be divided into two groups: one group includes the *ram* locus (the *ramR-romA-ramA* genes) and the efflux pump *acrA*, and the other group includes *gsiA* and *entE*. The *gsiA* gene encodes an ATP-binding protein of a glutathione importer [26], and EntE is an enzyme involved in the enterobactin biosynthesis pathway [27].

**Table 3:** Differentially expressed genes in laboratory-evolved strains compared with wild-type and clinical strains.

Gene	Gene	Product	XH210	XH211	XH212	XH213	XH214	XH215	XH216
up-regulated									
LQ47_01505		phospholipid ABC transporter substrate-binding protein	2.2*	4.0	4.3	3.5	3.4	3.5	3.8
LQ47_01510		ABC transporter substrate-binding protein	2.1*	4.0	4.2	3.9	3.1	3.6	4.1
LQ47_01515		phospholipid ABC transporter substrate-binding protein	2.8*	4.5	4.7	4.5	4.1	4.5	4.9
LQ47_08715		nitrate/nitrite sensor protein NarX	-0.5	3.0	2.9	3.5	3.0	2.7	2.9
LQ47_08720		nitrate/nitrite transporter NarK	-1.1	6.2	4.9	5.5	4.9	5.2	5.9
LQ47_08730		nitrate reductase	-0.7	5.1	4.5	5.2	4.6	4.3	5.1
LQ47_08735		nitrate reductase	-0.3	5.3	4.5	5.5	4.8	4.3	5.3
LQ47_08740		nitrate reductase	-1.0	4.4	4.3	4.9	4.9	3.9	4.8
LQ47_08745		nitrate reductase	2.6*	7.1	6.6	7.1	6.7	6.5	7.3
LQ47_16215		sensor protein BasS/PmrB	1.9*	4.0	3.3	4.2	3.9	3.8	4.0
LQ47_22580		transcriptional regulator	3.6*	4.9	6.4	5.0	4.9	5.1	5.6
down-regulated									
LQ47_02160		hypothetical protein	-1.1*	-10.4	-6.8	-5.9	-6.7	-7.4	-8.9
LQ47_04290		hydrogenase 3 membrane subunit	-0.5	-4.9	-4.1	-6.3	-6.8	-5.9	-4.7
LQ47_04295		hydrogenase 3 large subunit	-0.2	-5.2	-4.5	-6.2	-6.7	-5.9	-4.9
LQ47_04310		formate hydrogenlyase maturation protein HycH	-0.6	-5.5	-3.7	-6.2	-5.9	-6.1	-4.6
LQ47_04315		hydrogenase 3 maturation protease	-0.2	-6.8	-5.5	-5.6	-10.9	-10.4	-5.6
LQ47_04680		fimbrial protein	0.1	-5.5	-8.2	-7.1	-5.7	-10.8	-7.1
LQ47_09405		formate dehydrogenase	-0.3	-5.8	-4.0	-5.2	-5.4	-4.7	-5.0
LQ47_09545		acetoin reductase	-0.5	-7.5	-4.8	-7.5	-6.4	-7.8	-7.1
LQ47_09550		acetolactate synthase	-0.4	-6.3	-4.5	-6.3	-7.9	-6.7	-5.3
LQ47_11630		hypothetical protein	-3.9*	-5.1	-10.3	-7.7	-10.3	-6.2	-7.8
LQ47_12010		methionine synthase	-2.4	-5.4	-8.5	-5.1	-7.4	-6.3	-6.0
LQ47_23900		5,10-methylenetetrahydrofolate reductase	-1.5	-4.8	-5.7	-6.0	-4.6	-6.1	-5.2
Common									
LQ47_04775	*acrA*	acriflavin resistance protein AcrA	14.9	19.3	14.3	15.1	17.5	15.3	16.8
LQ47_10655	*gsiA*	glutathione ABC transporter ATP-binding protein	16.4	6.3	6.2	7.0	6.9	13.4	14.6
LQ47_17285	*entE*	enterobactin synthase subunit E	10.6	16.5	17.2	10.2	10.8	19.9	18.2
LQ47_17585	*ramA*	transcriptional regulator	5.2	6.3	5.3	7.1	5.7	6.8	8.2
LQ47_17590	*romA*	beta-lactamase	6.4	14.3	15.2	13.6	14.1	6.8	8.0
LQ47_17595	*ramR*	TetR family transcriptional regulator	4.9	3.6	5.1	4.9	6.0	4.3	4.9
LQ47_22005		membrane protein	4.2	3.8	5.1	5.3	3.6	5.3	4.9

*: differentially expressed genes in XH210, clinical isolate.

**Fig 2:**
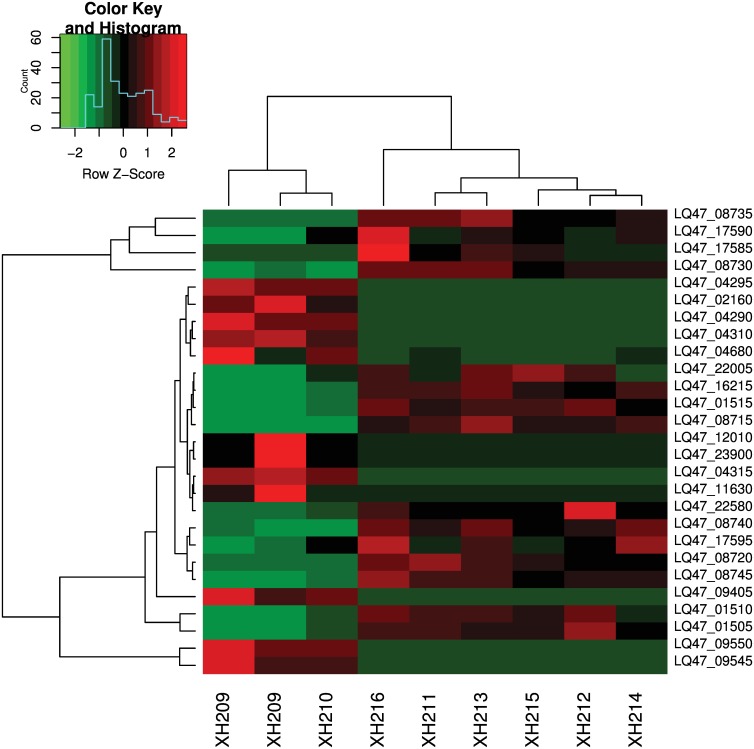
Heatmap of differentially expressed genes in the laboratory-evolved strains but not in the clinical strains.

**Fig 3:**
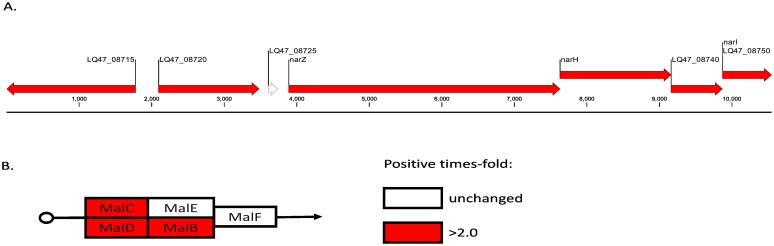
Comparison of the transcriptional profiles of genes in the laboratory-evolved strains with those of the wild-type and clinical strains. A). Changes in the transcription of genes involved in nitrogen metabolism between the laboratory-evolved strains and the wild-type and clinical strains. B). ABC transporters were induced in the laboratory-evolved strains. All values show the fold-change differences. The genes depicted in white were not differentially regulated.

To investigate differences between the clinical and the laboratory-evolved strains, we selected genes that were differentially expressed in the laboratory-evolved strains but not in the clinical strains (Fig 2). A total of 23 genes were differentially expressed only in the laboratory strains, and these included 11 up-regulated and 12 down-regulated genes (Table 3). After mapping the genes to pathways, we found that several genes involved in nitrogen metabolism were up-regulated (Fig 3A). In addition, ABC transporters were also induced (Fig 3B).

### Mutation in *ramR* was dominant in the single-step tigecycline resistance evolution experiment

**Fig 4:**
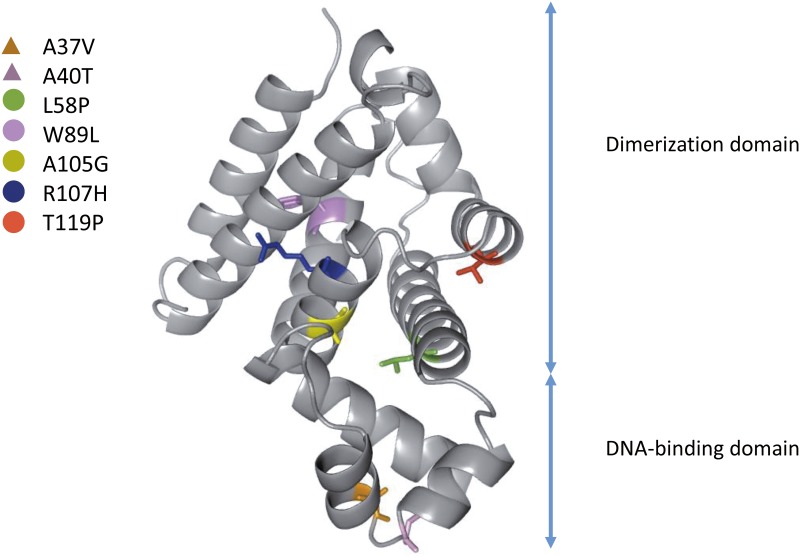
Homology modeling of *K*. *pneumoniae* RamR. The mutation sites are mapped onto the structure of RamR, and the amino acids are labeled.

Twenty mutants were obtained through single-step evolution experiments. The Sanger sequencing results for the *ramR* gene in the mutants obtained from the single-step evolution experiments showed that 80% (16/20) of the strains harbored a mutation in *ramR*, including base substitutions, frameshifts, insertions and deletions (Table 2). The tigecycline MICs of the mutants ranged from 8 to 16 mg/L, and the fitness costs ranged from 1 to 12%. Notably, only one strain showed a fitness cost of 12%, whereas the fitness costs of the other strains were not greater than 3%. The structure of *K*. *pneumoniae* RamR was subjected to homology modeling using the SWISS-MODEL workspace, and the mutation sites in the structure were labeled (Fig 4): five mutations were found to be located in the dimerization domain, and two mutations were localized in the DNA-binding domain.

### Tigecycline-resistant mutants showed cross-resistance to other antibiotics

To test the influence of the mutations on the response of these bacteria to other antibiotics, six different antibiotics belonging to several major classes (tetracycline, chloramphenicol, amikacin, cefotaxime, ciprofloxacin and imipenem) were tested (Table 2). The MICs of the mutants for ciprofloxacin increased from 0.75 mg/L to 2–6 mg/L, which might be caused by up-regulation of an efflux pump gene, *acrA*. Furthermore, XH211 became sensitive to beta-lactams, including cefotaxime and imipenem.

### The role of *ramR* and *lon* in tigecycline resistance was confirmed by complementation and gene knockout

To confirm the roles of *ramR* and *lon* in tigecycline resistance, we cloned the wild-type *ramR* and *lon* genes into the pCR2.1-T vector and introduced the plasmids into the tigecycline-resistant mutants. Tigecycline sensitivity was restored in the XH210 strain carrying the *ramR* plasmid but not in bacteria carrying the empty vector. Analysis of the *in vitro* selection mutants revealed that the plasmid carrying *ramR* or *lon* only partially restored sensitivity to tigecycline (Table 4). It should be noted that the resistant mutant that presented only a partial restoration of sensitivity after transfection of the plasmid harboring both *ramR* and *lon* showed mutations in other genes, such as *ramA*, *rpsJ* and *rpoC*. This result might indicate the involvement of *ramA*, *rpsJ* and *rpoC* in tigecycline resistance.

**Table 4:** Complementation experiment.

TGC MIC (mg/L)		Wild type	pCR2.1-T vector	pCR2.1-ramR	pCR2.1-lon	pCR2.1-lon-ramR
Strain	Genotype					
XH210	ramR Q122*	8	8	4		
XH211	ramA Q72L lon Q317* ramR Δ190 bp (322–511)	128	64	16	4	16
XH212	rpsJ V57L lon D445V PramR +G	256	256	256	128	64
XH213	ramR A40T lon R33W rpoC Δ18 bp (634–651)	128	128	4	64	32
XH214	ramR L58P rpoC G336A	64	64	8		
XH215	ramR Q135* lon Δ9 bp (791–799) rpoC S263Y	256	128	8	16	32
XH216	ramR S29* lon N417K	64	64	32	64	32

Note:

*: stop codon;

Δ: deletion; bp: base pair; NA: no mutation.

The roles of *ramR* and *lon* in tigecycline resistance in *K*. *pneumoniae* were also verified by gene knockout. The *ramR* and *lon* genes were knocked out in the XH209 strain, and the resulting mutants displayed higher tigecycline resistance than the wild-type strain, although the tigecycline MIC of the *ramR* mutant was higher than that of the *lon* mutant (Table 5). Moreover, the relative growth rate of the *ramR* and *lon* mutants were measured, and both showed slower growth in MH medium compared with the wild-type strain.

**Table 5:** Tigecycline MICs and relative growth rates of *K*. *pneumoniae* XH209 and its isogenic mutants.

Strain	Genotype	TGC MIC (mg/L)		Relative growth rate
		Broth	E-test	
XH209	wt	2	1	100.0
XH872	Δ*ramR*::apr	16	12	93.6
XH889	Δ*lon*::apr	8	3	96.3

## Discussion

In this study, we found that the MICs for tigecycline increased in a step-wise manner with the presence of mutations in the *ramR* operon and the *lon* and *rpsJ* genes. Our transcriptional analysis results showed that the *ramR* operon is highly expressed in all seven tigecycline-resistant *K*. *pneumoniae* strains, indicating that the *ramR* operon plays an important role in tigecycline resistance in *K*. *pneumoniae*. The *ramR* gene, located upstream of *ramA*, encodes a transcriptional repressor belonging to the TetR family, and a mutation in *ramR* leads to the overexpression of *ramA* [28, 29]. This regulation is achieved via the binding of RamR to the promoter of *ramA* [30]. Nonsynonymous mutations in *ramR* are reported with high frequency in tigecycline-non-susceptible *K*. *pneumoniae* clinical isolates [7]. We also identified base substitutions, insertions and deletions in the *ramR* gene (7/7) in *K*. *pneumoniae*, confirming these previous findings. These results indicate that *ramR* mutation is a common mechanism involved in tigecycline resistance.

The Lon protease is involved in the degradation of MarA in *Escherichia coli* [31]. A loss-of-function mutation in *lon* would lead to higher concentrations of MarA, which would increase expression of the AcrAB efflux pump. We detected three different types of point mutations in the *lon* gene, and complementation and gene knockout experiments demonstrated that *lon* mutants exhibited higher resistance to tigecycline than wild-type *K*. *pneumoniae*. Inactivation of *lon* is involved in the mechanism of tigecycline resistance in *E*. *coli* and *S*. *Typhimurium* [32, 33]. To the best of our knowledge, this study includes the first construction of a *lon* mutant in *K*. *pneumoniae*, which allowed confirmation of the association of mutations in this gene with tigecycline resistance. A transcript analysis showed that XH211, XH212, XH215 and XH216 presented higher expression levels of *oqxAB* compared with the wild-type strain. These results suggest that RarA and OqxAB play an important role in laboratory-evolved tigecycline-resistant strains [34], whereas the expression of *oqxAB* might be regulated by *lon* in all four strains that harbor *lon* mutations.

RpsJ is thought to act as a general target of tigecycline adaption and a marker for alterations in antibiotic resistance in bacteria [35]. The protein encoded by the *rpsJ* gene is a component of the 30S ribosomal subunit and participates in the formation of a BoxA-binding module [36]. Villa *et al*. reported an amino acid substitution of V57L in *K*. *pneumoniae rpsJ* [8], and our results confirmed the presence of this amino acid substitution in this gene. The V57L mutation might cause weaker binding of tigecycline to 16S rRNA, leading to tigecycline resistance [8]. The S10 mutation has also been reported in *Enterococcus faecium*, *E*. *coli*, *Staphylococcus aureus*, *Streptococcus pneumoniae* and *Acinetobacter baumannii* [35, 37, 38]. However, we did not achieve *rpsJ* knockout in *K*. *pneumoniae*. In addition, all attempts to achieve allelic replacement at this locus in *E*. *coli*, *A*. *baumannii* and *E*. *faecium* have failed [35, 39]. This failure could be due to the essential role of S10 in translation and transcription.

Overall, the dominant genetic mutations associated with tigecycline resistance in *K*. *pneumoniae* were found in the *ramR*, *lon* and *rpsJ* genes. Furthermore, the *ramR* locus was found to be highly expressed in all tigecycline-resistant strains. A higher fitness cost was observed in the laboratory-evolved strains but not in the clinical strains. We found differences in the transcriptional changes between the laboratory-evolved tigecycline-resistant mutants and the clinical tigecycline-resistant isogenic strains. Complementation experiments and knockout construction confirmed the roles of *ramR* and *lon* in tigecycline resistance in *K*. *pneumoniae*. We believe that we are the first to construct a *lon* mutant in *K*. *pneumoniae*, which allowed us to confirm its association with tigecycline resistance. These results suggest that the *ramR* operon and the *lon* and *rpsJ* genes play central roles in tigecycline resistance in *K*. *pneumoniae*.

## Nucleotide Sequence Accession Numbers

The nucleotide sequences of XH209 have been deposited at DDBJ/EMBL/GenBank under the accession number CP009461. The whole-genome shotgun sequencing results for XH210, XH211, XH212, XH213, XH214, XH215 and XH216 have been deposited at DDBJ/EMBL/GenBank under the accession numbers JUGC00000000, JTEA00000000, JTEB00000000, JTGO00000000, JTJA00000000, JUBD00000000 and JUBE00000000, respectively.

## Supporting Information

S1 Table Primers used in this study. (DOCX)
