# Supplementary material for: Step-Wise Increase in Tigecycline Resistance in Klebsiella pneumoniae Associated with Mutations in ramR, lon and rpsJ
Source: PLoS One. 2016 Oct 20;11(10):e0165019. doi: 10.1371/journal.pone.0165019 (PMC5072711; doi:10.1371/journal.pone.0165019)
Supplement: S1 Table — (DOCX) [file pone.0165019.s001.docx]

S1 Table: Primers used in this study

| Gene | Primer | Sequence (5'->3') |
| --- | --- | --- |
| *rpoB* | 7-1-R2 | GGATCCTGAGACGGAACGATGATTGG |
|  | 7-1-F2 | GGATCCAAAGATTATGAGTGCCTGTGCG |
| *rpsJ* | 22-1-R2 | GGATCCCAATCGTAATGGGTATGAGGAG |
|  | 22-1-F2 | GGATCCTAACACGGTTTGCTTCAACTT |
| *lon* | 4-2-R2 | TCTAGACAAGGTCGCGTTTGTTCTCAT |
|  | 4-2-F1 | TCTAGAGCTGGTTCGCACCGCTATCA |
| *ramR* | 4-1-R2 | TCTAGAGAGACGCTTCCACCTGGCTAA |
|  | 4-1-F2 | TCTAGACGGGTTCGATACTGCGATAAATT |
| *tetA* | 38-1-R2 | GGATCCGGCGGCACGGATCACTGTATT |
|  | 38-1-F2 | GGATCCACGCTGAGTGCGCTTCAAATC |
| *ramA* | 4-3-F1 | GATTTATTACAGCGGCGACAGC |
|  | 4-3-R1 | CGTTAAGCATTATTTTACCGGACAA |
| *ramR* knockout | ramR-D-F1 | TAAACGGGTAGGTCAGGGCGATACGGTGAGCGCAGGGATGCAGTGTTTCCGGCGTCATTAtgtaggctggagctgcttc |
|  | ramR-D-R1 | CCTGGTCAGACGTGCCAAGATCGGCGGTTTGTTTAAACCTGCGTGAGGAAAAAAGTAGTGattccggggatccgtcgacc |
| *lon* knockout | lon-D-F1 | ATGGTCATACCCCTGTTCGTAGGGCGGGAAAAATCTATCCGTTGCCTCGAAGCGGCCATGattccggggatccgtcgacc |
|  | lon-D-R1 | GCAAGTCCGAAATGACTTACCAGCCCTATTTTTATTAGCGCAATTTGCGCAGAATCACTAtgtaggctggagctgcttc |
